# The promise of molecular science in brain health. What breakthroughs are anticipated in the next 20 years?

**DOI:** 10.1016/j.cccb.2024.100364

**Published:** 2024-08-22

**Authors:** Atticus H Hainsworth, Thomas P Blackburn, Elizabeth M Bradshaw, Fanny M Elahi, Philip B Gorelick, Jeremy D Isaacs, Anders Wallin, Steven CR Williams

**Affiliations:** a Molecular & Clinical Sciences Research Institute, St George's University of London, London, SW17 0RE, UK; b Department of Neurology, St George's University Hospitals NHS Foundation Trust, Blackshaw Road, London, SW17 0QT, UK; c Translational Pharmacology BioVentures, Leigh on Sea, Essex, SS9 2UA, UK; d TPBioVentures, Hoboken, NJ, USA; e Carol and Gene Ludwig Center for Research on Neurodegeneration, Department of Neurology, Columbia University Irving Medical Center, New York, NY, USA; f Departments of Neurology and Neuroscience, Ronald M. Loeb Center for Alzheimer's Disease, Friedman Brain Institute, Icahn School of Medicine at Mount Sinai, 1 Gustave L. Levy Place, New York, NY 10029-5674, USA; g James J. Peter VA Medical Center, Bronx, NY, USA; h Davee Department of Neurology, Northwestern University Feinberg School of Medicine, 635 N. Michigan Avenue, Chicago, IL 60611, USA; i Department of Psychiatry and Neurochemistry, Institute of Neuroscience and Physiology, Sahlgrenska Academy, University of Gothenburg, Mölndal, Sweden; j Department of Neuroimaging, Institute of Psychiatry, Psychology & Neuroscience, Kings College London. SE5 8AF, UK

**Keywords:** Brain health, Neuroscience, Brain function, Brain disease, Ageing

## Abstract

Brain health means optimal physiological brain function across the normal life-course. It encompasses not only healthy brain aging but also brain diseases, their diagnosis and treatment. In all these areas, molecular science has advanced our understanding.

This multi-disciplinary review combines viewpoints from laboratory science, clinical medicine and the bioscience industry. First, we review the advances that molecular science has brought to brain health in the past twenty years. These include therapeutic antibodies for CNS diseases (multiple sclerosis, Alzheimer disease) and the dramatic introduction of RNA-targeted therapeutics. Second, we highlight areas where greater molecular understanding is needed. Salient examples are the relation of brain structure to cognitive symptoms, and molecular biomarkers for diagnosis, target discovery and testing of interventions. Finally, we speculate on aspects of molecular science that are likely to advance brain health in the next twenty years. These include: cell senescence and chronobiology; gene editing (notably, CRISPR) and RNA targeting (RNA interference, miRNA manipulation); brain-immune interactions; novel drug targets (AQP4, HIF1, Toll-like receptors); and novel chemistry to make new drugs (molecular machines, quantum molecular modelling and “click” chemistry). Early testing of the relationships between molecular pathways and clinical manifestations will drive much-needed breakthroughs in neurology and psychiatry.

## Highlights

• In the past twenty years molecular science has delivered progress in therapeutic antibodies, biochemical markers, gene therapy and drug re-purposing.
• Areas of need relevant to brain health include: brain development and plasticity of neuronal circuits; more refined molecular biomarkers for diagnosis and as clinical trial endpoints; better understanding of the relation of brain structure to cognitive disease.
• The next twenty years is likely to witness progress in: gene editing and RNA targeted interventions; manipulating cell fate; brain-immune interactions; new classes of drugs, derived from novel chemistry.

## The 20-year perspective

Looking ahead to the next 20 years feels immeasurably remote, mysterious and, in view of the existential threats from climate change, human aggression and the next pandemic, somewhat forbidding. At the time of writing, looking back 20 years takes us to 2003. That doesn't sound so very far back. How was the World back then? The animated fable “*Finding Nemo*” was grossing in cinemas worldwide. The US and UK had declared war on Saddam Hussein's Iraq. And no-one had an iPhone (they would have to wait 4 more years).

In this paper we will assess the main contributions of molecular science (so called “Basic Science”) to brain health in the past 20 years, and the current areas of salient need. We will then attempt to predict the contributions that will translate from molecular science over the next two decades.

## What has basic science done for brain health in the past 20 years?

Brain health may be defined in broad or narrow terms. Simply put, optimal brain health is the preservation of brain structure and function across life's course [1]. Whereas brain health may be focused on a specific epoch of life, it is continuous across the entire lifespan and is influenced by factors that may be taken for granted (e.g., food security, social isolation, status of mental health, climate, and political upheaval). Many of the threats to brain health such as cardiovascular risks are modifiable but may require a convergence of sciences, public health policies, and government prioritization to prevent brain injury and maintain brain health on a global scale. In this review we focus on the molecular aspects of brain health, particularly in relation to brain vascular disease (reflecting the title of this journal). The paper is part of a series of articles on Brain Health, edited by three of the present authors (Gorelick, Hainsworth, Wallin) [2].

It was claimed that cataloguing the DNA sequence of the wild-type human genome (published in 1998) would revolutionize medical science, including brain science. Though slow, this promise has begun to be realized (see Table 1). Improvements in the efficiency of DNA sequencing permit the coding regions of one's entire genome to be sequenced within a few days. More importantly for the clinician, a specific gene or cluster of genes can be sequenced, with confidence and precision, within a similarly rapid timescale. Twenty years ago we could analyse individual genes for a particular neurological diagnosis (e.g. PRNP, Huntingtin). Now, the physician does not have to choose specific genes, but can request a panel of genes of interest, following whole exome or whole genome sequencing. This means that our power to find pathogenic variants has increased significantly, and makes is easier to identify genetic forms of disease and counsel patients and families accordingly. Importantly, novel analytical methods are moving conceptualization of disease from monogenic searches to polygenic risk for phenotypic traits. It should be noted that aside from rare single genetic disorders with high penetrance, most genetic variants are too weakly associated with their target disorders to be of clinical value at present. Currently, in the majority of cases genotyping does not result in treatment, although the list of treatable neurogenetic disorders is increasing (examples being acid maltase deficiency, ATTR amyloid, SMA, SOD-ALS).

**Table 1:** What has molecular science done for brain health in the past 20 years?

Advance	Example(s)
Gene therapy	infant ataxias, including SMA
Drug re-purposing	tPA for ischemic stroke; thalidomide in neuro-oncology; ketamine in major depressive disorder
Biochemical markers for detection of brain pathologies in living humans	p-Tau217, etc. in AD. NF-L in AD and related dementias
Therapeutic antibodies and other immune modulators	Tysabri™ for MS Aducanumab, Lecanumab, Donanemab for AD

Abbreviations: AD: Alzheimer disease; MS: multiple sclerosis; SMA: spinal muscular atrophy.

Development of new drugs in the field of clinical neurology is notoriously slow. Nevertheless the past twenty years have seen a significant trend in the use of therapeutic antibodies for treating CNS diseases. The ICAM-1 antibody natalizumab (Tysabri™) has been a major advance in the treatment of Multiple Sclerosis [3,4]. More recently, therapeutic antibodies designed to remove beta-amyloid deposits from the brain, have been licensed for the treatment of Alzheimer's type of major cognitive impairment [5,6]. The next 20 years will give clarity on these antibody treatments and offer ways around amyloid-related imaging abnormalities (AIRA) which are currently a potentially serious complication of the treatment [7].

**Fig. 1:**
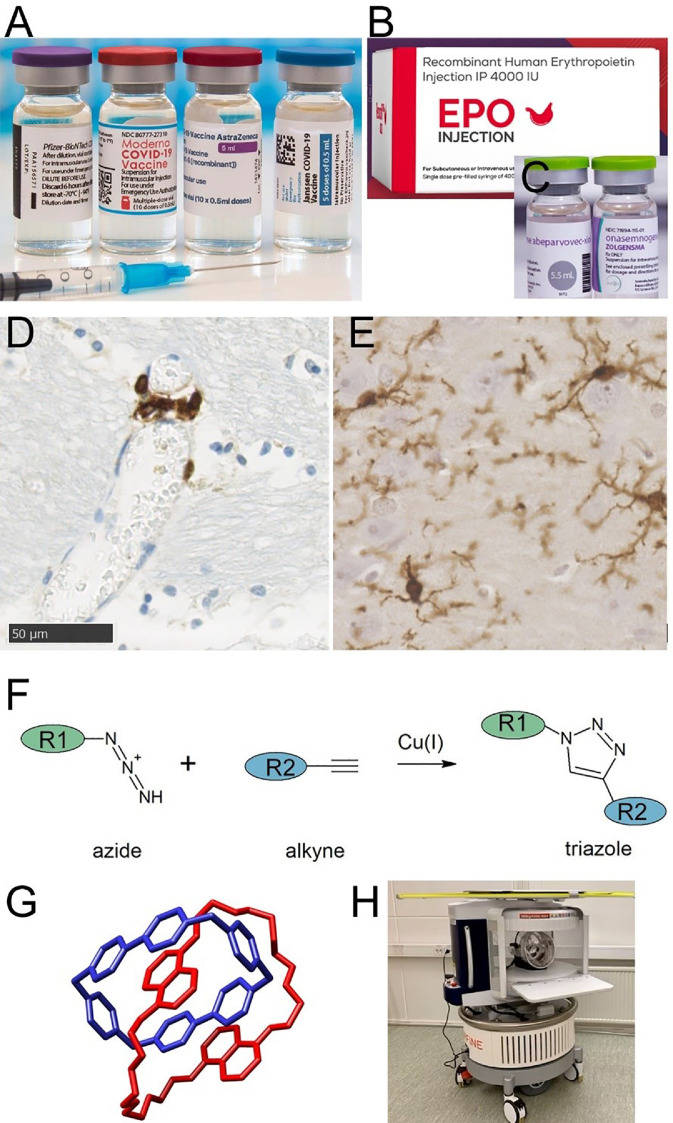
A-C) medicines resulting from recent molecular science. A) SARS-COVID-19 vaccines, containing RNA encoding a viral antigen. B) recombinant erythropoietin. C) a formulation of antisense oligonucleotide, designed to silence a disease-causing mutation. D-E) immune cells relevant to brain health. D) extravascular T-cells, around a small blood vessel in human brain tissue. T-cells are immunolabelled with CD3 (brown). Haematoxylin nuclear counterstain (blue). E) Resident microglia in brain tissue, immunolabelled with IBA-1 (brown). F-H) likely areas of future progress. F) Example of Click Chemistry. Two biological entities, R1 and R2, are bonded in a Cu(I) catalysed reaction. G) Example of a molecular machine. The interlocked molecule cyclobis-(paraquat-p-phenylene) or “catenane”, synthesized by Stoddart and coworkers. H) Ultra-low field MRI scanner, running on mains voltage and without requirement for liquid helium.

The SARS-2-COVID-19 pandemic gave enormous impetus to vaccine development. Most noteworthy is the rapid and successful delivery of RNA-based vaccines, the first to market being produced by BioNTech-Pfizer (Fig. 1A). Treatment with a small synthetic RNA encoding an antigen, rather than the antigen itself, is a landmark in disease prevention (recognised in the 2023 Nobel prize for Physiology and Medicine). This strategy was a major factor in managing COVID-19. Use of RNA-based immunogens is now likely to extend to neurological conditions.

More prosaically, the concept of repurposing existing drugs from other indications has gained in popularity in brain health. The thrombolytic agent tissue plasminogen activator (tPA) was borrowed from the field of cardiology. Though the use of tPA in acute ischemic stroke was licenced a few years before 2003, its main impact has been felt within the last 20 years, entering routine stroke care and affording radical change to acute stroke services, and to the prospects of stroke patients. Another class of drugs, the HMG-CoA-reductase inhibitors (or statins) revolutionized prevention of coronary heart disease as early as the late 1980s and was eventually shown to have application for prevention of atherosclerotic stroke [8]. Other examples of drug repurposing include thalidomide for cancer treatments (including CNS cancers) and ketamine for the treatment of chronic depression.

The molecular biology revolution has led to rapid advances in biotechnology methods for engineering DNA and RNA molecules, with some evidence of translation already appearing in clinical medicine. The antisense oligonucleotide nusinursen targets pre-mRNA splicing of the *SMN2* gene, giving clinically meaningful benefits in the childhood ataxia, spinal muscular atrophy (SMA; Fig. 1C) [9]. Similarly, antisense silencing of *FUS* expression has shown benefit in amyotrophic lateral sclerosis (ALS) [10]. In Huntington's disease, an adult-onset autosomal dominant disorder, an antisense strategy has been less successful, despite considerable promise in early phase clinical trials [11]. We anticipate major advances in this area over the next 20 years, with application in other aspects of brain health.

## What are the biggest molecular “needs” in brain health?

Clinical approaches to brain health are based on structure and anatomy. MRI brain scanning, the workhorse of clinical neurology, reports the density, distribution and environment of a single small molecule, H_2_O. Equally rooted in structure and function are the readouts from the EEG monitor, the clinical history of cranial nerve reflexes and reporting of neurological symptoms, all with little molecular detail. Diagnostic neuropathology reports on vacuolation, cellular swelling, greater or lower density of microglial cells, or of axons, or some other structural feature. Immunohistochemistry can provide highly specific molecular mapping (for example, of certain subtypes of neurofilament) [12] but these are employed in diagnosis surprisingly rarely. In the era of 21st century biology one might wonder at this lack of molecular refinement. We list some top-line areas of need in Table 2.

**Table 2:** What are the molecular science “needs”, the gaps in vascular brain health?

Relation of brain structure and neuroanatomy to cognitive disease, specifically to vascular dementia and vascular cognitive impairment (VCI)
Understanding brain development, brain axonal wiring and plasticity of brain circuits
Molecular readouts to link brain imaging features to molecular and cellular brain pathology
Molecular biomarkers for better diagnosis, for discovering therapeutic targets and as quantitative endpoints in clinical trials
Predictive animal models relevant to human brain vascular disease and brain aging

These are not listed in any significant order.

An area notably ripe for growth is that of molecular biomarkers, especially blood-based biomarkers. Methodological advances have led to faster throughput, smaller sample volumes (less than 0.1 millilitre) and multiplexed analytes (notably, using SIMOA platforms). Tangible progress has been seen in recent years in the AD field, with antibody-based blood assays of particular phosphorylated residues in the tau protein, and of the neurofilament NF-L [13,14]. Progress with synthetic antibodies (for example, AI-designed binding sequences) for analyte detection are likely to fuel this advance. Non-protein biomarkers are beginning to emerge. These include molecular species derived from on-going efforts in large cohorts, using serum/plasma/CSF lipidomics and metabolomics in Parkinson's disease and ALS [15,16].

Robust molecular biomarkers have enormous potential, not only for refining diagnoses and screening at-risk populations, but also as quantitative endpoints for testing interventions in clinical trials. A recent example is the alpha-synuclein seed amplification assay, recently validated in antemortem CSF samples from a neuropathologically confirmed cohort of LBD cases [17]. There is considerable merit in a molecular analyte (or more likely, a panel of them) which are likely to be more quantitative - and less costly - than a specialised imaging modality or an expert clinical assessment.

## What will molecular science do for brain health in the next 20 years?

Predicting the future is not easy. Looking for medical breakthroughs in the next 20 years, one might do worse than scan the Nobel prizes awarded in the past 20 years (Table 3). There we see trends for understanding whole chapters of novel cell biology. These include the Yamanaka factors that govern cell differentiational fate, and ubiquitin-dependent trafficking that recycles proteins within the cell interior. In addition there are novel molecular pathways and potential therapeutic targets, including the Toll-like receptors, aquaporin-4 in astrocyte endfeet, and the hypoxia-sensing factor HIF1α. There are also new, ingenious ways to visualise sub-cellular drama, including super-resolution microscopy and cryo-electron microscopy.

**Table 3:** Nobel prizes for physiology & medicine and for chemistry 2002–2023.

Year	Physiology & Medicine Laureates	Physiology & Medicine Subject Area	Chemistry Laureates	Chemistry Subject Area
2002	Sydney Brenner (South Africa/UK) H. Robert Horvitz (USA) John E. Sulston (UK)	Genetic regulation of organ development and programmed cell death	John B. Fenn (USA) Tanaka Koichi (Japan) Kurt Wüthrich (Switzerland)	NMR and mass spectrometry techniques, to analyze proteins and other large molecules
2003	Peter Mansfield (UK) Paul Lauterbur (USA)	Magnetic resonance imaging (MRI)	Peter Agre (USA) Rod MacKinnon (USA)	Aquaporins and X-ray crystal structure of ionic channels
2004	Linda B Buck (USA) Richard Axel (USA)	Odorant receptors and organization of the olfactory system	Aaron Ciechanover (Israel) Avram Hershko (Israel) Irwin Rose (USA)	Ubiquitin-mediated protein degradation
2005	Barry J. Marshall (Australia) J. Robin Warren (Australia)	Helicobacter pylori and its role in gastritis and peptic ulcer	Yves Chauvin (France) Robert H. Grubbs (USA) Richard R. Schrock (USA)	Metathesis, a novel, non-polluting form of organic synthesis (“green chemistry”)
2006	Andrew Z. Fire (USA) Craig C. Mello (USA)	Gene silencing by RNA interference (RNAi)	Roger D Kornberg (USA)	Molecular basis of genomic DNA transcription
2007	Mario R. Capecchi (Italy/USA) Martin J. Evans (UK) Oliver Smithies (UK/USA)	Transgenic mice, derived from genetically altered embryonic stem cells	Gerhard Ertl (Germany)	Surface chemistry at gas-solid interfaces
2008	Harald zur Hausen (Germany) Françoise Barré-Sinoussi (France) Luc Montagnier (France)	Discovery of human papilloma viruses causing cervical cancer, and of HIV	Martin Chalfie (USA) Osamu Shimomura (USA) Roger Y. Tsien (USA)	Green fluorescent protein, GFP
2009	Elizabeth H. Blackburn (USA/Australia) Carol W. Greider (USA) Jack W. Szostak (USA)	Chromosomal telomeres and the enzyme telomerase	Venkatraman Ramakrishnan (USA) Thomas Steitz (USA) Ada Yonath (Israel)	Structure and function of the ribosome
2010	Robert G Edwards (UK)	In vitro fertilization	Richard F. Heck (USA) Negishi Ei-ichi (USA) Suzuki Akira (Japan)	Palladium-based catalysis to synthesize complex organic molecules
2011	Bruce A. Beutler (USA) Jules A. Hoffmann (France/Luxembourg) Ralph M. Steinman (Canada)	Role of Toll-like receptors, and dendritic cells, in innate immunity	Daniel Shechtman (Israel)	Quasicrystals in metal alloys, with symmetrical but non-repeating atomic structure
2012	John B. Gurdon (UK) Shinya Yamanaka (Japan)	Reprogramming of mature cells to become pluripotent, via a small number of transcription factors (“Yamanaka factors”)	Brian K. Kobilka (USA) Robert J. Lefkowitz (USA)	Structure and function of G-protein-coupled receptors (GPCRs)
2013	James E. Rothman (USA) Randy W. Schekman (USA) Thomas C. Südhof (Germany/USA)	Vesicular trafficking within cells	Martin Karplus (Austria/USA) Michael Levitt (UK/USA/Israel) Arieh Warshel (Israel/USA)	Quantum molecular models for complex chemical systems
2014	John O'Keefe (UK/USA) May-Britt Moser (Norway) Edvard I. Moser (Norway)	Hippocampal “place” neurons, activated when an animal enters a specific location	Eric Betzig (USA) Stefan W. Hell (Germany) William E. Moerner (USA)	Super-resolution fluorescence microscopy
2015	William C. Campbell (Ireland/USA) Satoshi Ōmura (Japan) Tu Youyou (China)	Novel therapies against roundworm parasites, and malaria	Tomas Lindahl (Sweden) Paul Modrich (USA) Aziz Sancar (Turkey/USA)	Mechanisms of DNA repair
2016	Yoshinori Ohsumi (Japan)	Mechanisms for autophagy	Jean-Pierre Sauvage (France) J. Fraser Stoddart (UK) Bernard Feringa (Netherlands)	Design and synthesis of interlocking molecules (“molecular machines”)
2017	Jeffrey C. Hall (USA) Michael Rosbash (USA) Michael W. Young (USA)	Molecular mechanisms controlling circadian rhythms	Jacques Dubochet (Switzerland) Joachim Frank (Germany/USA) Richard Henderson (UK)	Cryo-electron microscopy for structure determination of biological molecules in solution
2018	James P. Allison (USA) Tasuku Honjo (Japan)	Inhibition of negative immune regulation (T-cell receptors, endogenous inhibitors)	George P. Smith (USA) Gregory P. Winter (UK) Frances Arnold (USA)	Phage display for directed evolution of antibodies; artificial evolution of enzymes
2019	Peter J. Ratcliffe (UK) William Kaelin Jr. (USA) Gregg L. Semenza (USA)	How cells respond to hypoxia (HIF1a, HIF1b, EPO)	John B. Goodenough (USA) M. Stanley Whittingham (UK/USA) Yoshino Akira (Japan)	Lithium-ion batteries
2020	Harvey J. Alter (USA) Michael Houghton (UK) Charles M. Rice (USA)	Discovery of Hepatitis C virus	Emmanuelle Charpentier (France) Jennifer Doudna (USA)	Clustered regularly interspaced short palindromic repeats-Cas9 (CRISPR) a method for genome editing
2021	David Julius (USA) Ardem Patapoutian (Lebanon/USA)	Receptors for Pain, heat and touch (including TRPV1)	Benjamin List (Germany) David W.C. MacMillan (UK/USA)	Asymmetric organo-catalysis to synthesize a particular optical isomer
2022	Svante Pääbo (Sweden)	Genomes of extinct hominins (e.g. Neanderthals) and association with COVID-19	Carolyn R. Bertozzi (USA) Morten P. Meldal (Netherlands) K. Barry Sharpless (USA)	Development of click chemistry and bioorthogonal chemistry
2023	Katalin Karikó (Hungary/USA), Drew Weissman (USA)	Nucleoside base modifications that enabled mRNA vaccines against COVID-19	Moungi G. Bawendi, Louis E. Brus, Aleksey Yekimov (all USA)	Discovery and synthesis of quantum dots

Information taken from: https://www.nobelprize.org/prizes/lists/.

Nationality given is the citizenship of the recipient at the time the award was made.

There are giant leaps forward in understanding cell fate and cellular aging. These include the recognition that cell senescence, autophagy and chronobiology are not features only of cells in culture on a Petri dish. They operate in the highly specialised cells of the brain, and we are learning the molecular pathways that control them. There are also major advances in manipulating gene expression at the single base level, including the physiological process of DNA repair, and the CRISPR-Cas9 system which can now be used to amend a short nucleotide sequence in genomic DNA. In Table 3 the column listing Chemistry prizes shows radically accelerated means for producing new drugs and reagents (in no special order, quantum molecular modelling, “click chemistry” and ‘phage display). And at the whole organism level there are molecular explanations of circadian rhythms, hippocampal neurones that register our sense of geographical “place”, and receptors that mediate how we perceive heat and pain. We anticipate that all of these have potential for improving brain health.

### Novel molecular pathways, new drugs

As novel molecular pathways are revealed, new drug targets emerge. As an example, aquaporins, AQP4 in particular, mediate water transport across cell membranes (see Table 3). This is particularly important in the brain, where osmotic cell swelling or shrinkage is disastrous for neuronal cells. Similarly, low oxygen challenge – hypoxia - is well known to be especially harmful to brain neurones. The cell biology of hypoxia-inducible transcription factors (HIFα, HIFβ) and their target genes, notably *EPO* encoding erythropoietin, is already translating to drug treatments (Fig. 1B). The aquaporins and the HIF system may offer druggable targets for improving brain health.

While aquaporins and HIFs have been known for decades, recent years have revealed a multitude of gene candidates, encoding potential drug targets, in “cell atlases” [18]. These are gargantuan listings of all the RNAs expressed at a detectable level in each cell within a tissue sample the size of a split pea. In 2009, the first single-cell RNA-sequencing paper was published [19] and that technology has continued to develop. Now single-nuclei (rather than single-cells) can be sequenced, allowing researchers to fully utilize historical samples, and at the same time evaluate open chromatin and sequence the transcriptome of individual nuclei. From brain samples, cell atlases have been compiled that reveal cell type-specific transcription patterns in various diseases including Alzheimer disease [20,21] and vascular malformations [18]. Humans struggle to analyse brain atlas data, but computers do it very quickly. This is an area (among many) where artificial intelligence (AI) is likely to aid diagnosis, predict response to treatment and accelerate short-listing of potential candidates as drug targets.

It seems likely that the coming decades will witness single-cell capabilities across other modalities, such as proteomics, metabolomics and the cellular infectome (lipidomics may be a greater challenge). Single cell proteomic catalogues - to match the RNA atlases - are emerging [22,23] and are likely to expand. These unbiased investigations describe cellular diversity and dysfunction, many aspects of which could translate into therapeutics, with the help of modern genetics [24] and AI [25]. The therapeutics will be only as good as the underlying science and the critical mass of expertly labelled data. Thus over the next 20 years we will continue this in-depth examination of genetic, transcriptomic, and biochemical diversity. It is an exciting prospect that these high-volume data streams will enable more personalized medicine, integrating sex, race and diverse ethnicities, as well as environmental factors.

### Cell fate

Cell senescence is a widespread cell biological phenomenon that has received considerable attention in the past 20 years (Table 3) [26]. This refers to an irreversible, non-replicative state that a cell can enter after multiple cell divisions. It is not a form of cell death, nor even of cell injury, and a senescent cell can live for decades. For example, a high proportion of brain vascular myocytes in an aged human brain are in a long-lived, stable senescent state [27]. Drugs that are claimed to be selectively toxic for senescent cells are being developed with the tempting title of “anti-senescence agents” [26,28]. A related field of cell biology is chronobiology, which refers to time-dependent processes and ageing in cells and organisms [29]. The concept of cellular age, distinct from chronometric age, has been a fertile idea farm. Bluntly expressed, two humans who have each lived for 80 calendar years might have very different biological ages. And there may be lifestyle factors, interventions and disease states that influence one's biological age [30]. Deeper understanding of the biology of cell aging and cell senescence hold out considerable potential for improving brain health [28]. This may be manifest in terms of genetic counselling and personalised medicine.

Autophagy and ubiquitination are also cellular phenomena relevant to cell fate. Each would now require a dedicated chapter in any Cell Biology textbook, though a few decades ago would barely have been mentioned. Autophagy (Table 3) is a physiological cytoplasmic process, in which redundant cellular components (organelles, membrane components) are trafficked for lysosomal degradation and re-use of their constituents, reflecting an aging cell's changing needs. Similarly, ubiquitination is a cytoplasmic tagging system that operates in all eukaryotic cells, to mediate protein recycling. Old or dysfunctional protein molecules are covalently bound by a small (8.6 KDa) ubiquitin protein. This labels them to be trafficked for degradation and re-use of their amino acid residues. Failure of either autophagy or ubiquitination causes cytoplasmic accumulation of cellular debris, which can lead to disease, particularly in long-lived cells such as neurons. In the sphere of brain health, defective autophagy is implicated in neurodegenerative diseases, including Huntington's disease and frontotemporal dementia [31]. Genetic mutations in the ubiquitination system cause severe childhood neurological diseases (an example being MNGIE) [32]. Neurological diseases of early life that result from mutations in the autophagy and ubiquitination pathways appear eminently suitable targets for gene therapy (gene delivery or RNAi), or some form of genetic manipulation (such as CRISPR).

Under this heading of Cell Fate, we recall as a reality-check another remarkable cell biology phenomenon: apoptosis, or programmed cell death (Table 3). Apoptosis promised to be a game changer in our understanding of brain health mechanisms, yielding new treatments for brain disease in the areas of neurodegeneration, stroke and traumatic brain injury [33]. Despite this, caspase inhibitors and other drugs to impede apoptosis have been disappointing, in that they have led to no new interventions for brain health.

Stem cells have also been heralded periodically as a source of hope for severe brain diseases. Stem cells were tolerated following surgical injection into brains of people who had suffered extensive stroke damage, with indications of some functional benefit [34]. In Parkinson's disease there is a long tradition of surgically implanting cells into midbrain nuclei, intended to replenish the depleted dopaminergic neurones [35]. In a recent phase-1 clinical trial dopaminergic neurone-like cells, derived from human embryonic stem cells, have been surgically delivered into the putamen of people with severe Parkinson's disease [36]. Incomplete understanding of the shifts in phenotypic identity that cells undergo, once released into a more-or-less intact human brain, may limit enthusiasm for these approaches.

### Manipulating gene expression

The process of RNA interference, which is a natural process in all eukaryotic cells (Table 3), is being harnessed for disease modification. This entails a small RNA sequence (typically 20 nucleotides) hybridising with its complementary sequence in a particular cellular mRNA, and thus marking it for enzymatic destruction. RNAi-based treatments are currently being prototyped in severe forms of peripheral cardiovascular disease. An example is the RNAi reagent inclisiran in metabolic syndrome and non-alcoholic hepatitis [37]. In patients with early onset AD, a current phase-1 clinical trial is testing an RNAi reagent targeting APP (https://clinicaltrials.gov/study/NCT05231785). It is tempting to speculate that the RNAi approach may have significant clinical impact in brain disease, though access to the target will remain a significant issue. RNAi has potential especially in dominant genetic disorders where a harmful mutant protein is the molecular cause (derived from just one allele, with the other allele normal and encoding functional protein)[24].

Staying in the realm of manipulating RNA biology, microRNAs (miRNA) are a portfolio of small, regulatory RNA molecules, encoded in the genome and present in all cells. Acting via complementary binding sites in genomic DNA, miRNA molecules augment or suppress expression of nearby loci. An example relevant to brain health is the monogenic brain vascular disorder PADMAL [38]. Here, intronic mutations alter the binding of miRNA-29 to its binding site within *COL4A1.* This large gene encodes alpha1 collagen type IV, an obligate component of the vascular basement membrane [38]. Coding mutations in *COL4A1* produce defective collagen alpha1-IV and a spectrum of vascular phenotypes, manifested primarily in brain vasculature [39]. The loss of miRNA-29 binding in PADMAL leads to excessive alpha1 collagen type IV, and a severe form of early-onset cerebral small vessel disease [38,40].

Gene editing techniques are highly likely to impact brain health in the next 20 years. The best known is CRISPR, rewarded with an all-female prize (Doudna and Charpentier 2020, Table 3) [41]. Harnessing molecular tools that have been discovered in the cell biology revolution, gene editing allows not only enzymatic deletion of one or a few selected bases from a genetic sequence, but also replacing them with another, selected base sequence. Understanding of the methodology and the accuracy of gene editing have improved over the past two decades. Coupled with developments in the growing field of stem cell technology, gene editing is likely to make considerable impacts in medical treatment of severe genetic diseases [42]. There is a clear opportunity for applying CRISPR approaches in monogenic diseases (such as HD, Motor Neurone Disease and familial AD). We predict that the next two decades will see a paradigm shift in gene therapies and cell therapies for brain health disorders.

### Brain-immune interactions

A role for immune cells, both resident microglia and infiltrating immune cells, is increasingly recognised in brain health. Immune cell involvement is well-appreciated for autoimmune CNS diseases such as multiple sclerosis, where the last 20 years have seen several immune-modulating drugs approved [4]. In other neurological disorders with a breach of the blood-brain barrier, such as stroke and traumatic brain injury, immune cells are involved in both tissue injury and repair [43,44]. Brain cancer, particularly glioblastoma, also manifests a complicated immune component [45]. Most notably, it is the neurodegenerative diseases where understanding has shifted from a dogma of neuron-centric disease process to a concept more dynamic, immune-neuron interactions underlying disease progression.

Toll-like receptors (TLRs, Table 3) are membrane glycoproteins with potent cell signalling actions, including activation of MAP kinases and NF-kB pathways [46]. Expressed in cells of the immune system (named after the Drosophila gene *Toll*) they have more recently been detected in brain cells, including neurons, oligodendroglia and microglia [47]. TLRs are activated by a wide variety of non-specific molecular signals that include microbial motifs (dsRNA is an example), lipopolysaccharides and damage-associated molecular patterns (DAMPs) in cell debris released from damaged or dying cells, sometimes at a substantial distance. These promiscuous signallers contrast with the lock-and-key specificity of peptide and protein pharmacology, seen for example in growth factors, hormones and their receptors. Nevertheless, in the brain-immune axis and in the brain-gut axis (whatever that turns out to be) TLRs are likely to be important molecular players, well-placed to influence brain health. Biomarkers and therapeutic agents targeted to the immune system already play a useful role in brain health and disease, and new discoveries will help these to expand further (TLR-related products, novel cytokines, endosomal vesicles).

Microglia were propelled to the centre of neurodegenerative disease investigation by the advent of genome-wide association studies (GWAS). From the first of these studies in late-onset Alzheimer's disease it was clear that genetics implicated the immune system, specifically the innate immune system [48]. As microglia are the resident innate immune cells of the brain (Fig. 1E), they have moved to the forefront of studies to determine molecular underpinnings of the genetic associations in AD. We are now looking at therapeutics being developed to specifically target microglia, and these genetically associated immune proteins (an example being TREM2) [49,50]. The next 20 years will determine the success of that strategy.

In other neurodegenerative diseases, large-scale genetic studies have not been as explicit in pointing to microglia. Even so, there are clear indications that a person's genetic background influences the immune system and its role in susceptibility to brain disease. One example is the association of the HLA genomic region with Parkinson's disease. This genetic connection of a chronic brain disease of older adults with antigen presentation is intriguing. It may be a clue to the gut-brain axis that is also implicated in Parkinson's disease [51,52]. Together, these associations impel us to look at brain-immune interactions more comprehensively and systemically, and to include all components of the immune system, even those cell types that are not highly present in the brain parenchyma, such as NK cells and T-cells (Fig. 1D).

### Brain-vascular interactions

The vessels of the brain develop in close association with the formation of the brain tissue. The interaction between the vessels and the brain parenchyma constitute the neurovascular unit (NVU) [53,54]. The close proximity between the brain cells and the vessels in the NVU implies that the traits of the brain vessels are different from those of other organs. The ability of the NVU to resist - or continue to sustain brain function despite - cerebrovascular lesions, proteinopathies, metabolic dysfunction and inflammation is likely a large factor in maintaining brain health. T he specific pathways are not yet understood, though the field is catching up (as testified by the formation of this journal, CCCB). There are also encouraging signs of increasing interest in biomarkers for brain vascular lesions [55].

### New drugs and reagents

It seems safe to predict a new era in drug discovery. RNA-based vaccines for neurological disorders hold promise of future application, as has been mentioned in an earlier section. Artificial intelligence (AI) has only just begun to reveal the extent of its power. AI is likely to have weaknesses – tasks for which it is ill-fitted – but it has strengths, where human intelligence is left far behind. Designing novel molecules to interact with endogenous molecules is likely to be such a strength. A more niche field than AI is quantum molecular modelling. Applying quantum mechanics - prototyped on the H_2_ molecule - to a biological protein is a daunting challenge, but this field has slowly and steadily advanced (Chemistry prize, 2013; Table 3) [56,57]. Using quantum mechanical computations of the individual atoms in small proteins (examples are pancreatic trypsin inhibitor, myosin head groups) led to descriptions of the fluid-like internal motions of the macromolecule, and verifiable predictions of biochemical activity. This advance has been in parallel with rising power of computer networks and availability of digital memory circuits.

There is also new chemistry. In “Click Chemistry” (Table 3) [58,59] small molecular building blocks are designed to form larger molecular structures, in high-yield organic reactions, under biological conditions [58]. Examples of the building blocks are a simple alkyne-azide mixture [59] (Fig. 1F). This process is analogous to the synthesis of macromolecules that is performed by any healthy cell, but without the requirement for dedicated, highly-evolved catalysts (i.e. enzymes).

Another, entirely new field, Molecular machines, has yielded molecules that are linked by physical interlocking, rather than by chemical bonds (Table 3) (Fig. 1G) [60]. These interlocked molecules have been used as physically contractile elements (described as “molecular muscles”, in reference to the actin-myosin interactions of biological muscle cells). One can envisage these molecular machines enabling novel biosensors (possibly in-dwelling ones) to report on biomarkers, or as smart drug delivery systems. The same chemistry is likely to yield a new generation of laboratory quantitation platforms (comparable to ELISA, RT-PCR or TaqMan).

Closer to conventional biochemistry, bacteriophage display is a high throughput method to detect protein-protein and protein-DNA interactions [61] and to produce “humanized” antibodies (Table 3). Phage display has already been used to develop new treatments for irritable bowel syndrome and rheumatoid arthritis (e.g. adalimumab). Another quantum leap from the biochemistry laboratory, artificial evolution is a clever way to speed up evolution in a test-tube (Table 3). It was developed in single-celled organisms to discover modified forms of proteins that function better than the naturally occurring ones.

These highly novel chemical and biochemical approaches will advance gene-targeted therapy, drug discovery and biomarker assay. The combination of artificial evolution with AI, combined with CRISPR and DNA repair (Table 3) will likely be a powerful one for discovering new molecular targets and possible therapeutics.

## What will brain imaging do for brain health in the next 20 years?

Whilst brain imaging technologies such as MRI continue to push the boundaries of sensitivity and specificity, this usually leads to more expensive and less accessible equipment. By marked contrast, recent advances in the development of ultra-low field MRI instruments (Fig. 1H) are cheaper than conventional scanners by a factor of 10 or more. These low energy, point-of-care scanners will revolutionize the scalability and access to brain imaging globally [62]. We envisage this simple, affordable technology will become universally used in management of acute events following stroke or traumatic brain injury. Once in place, the hardware will be used to acquire worldwide, inclusive and diverse Big Data on brain development (relevant to early life brain health) and neurodegeneration (in late life). Stratification and monitoring of emerging treatments for conditions such as multiple sclerosis and dementia will be commonplace beyond the confines of the radiology department [63].

With the advent of open science and greater willingness to share data, the integration of brain imaging information across modalities will dramatically increase our understanding of the underlying molecular systems in health, disease and in response to putative treatments. The recent introduction of comprehensive, brain-wide gene expression atlases such as the Allen Human Brain Atlas: (https://portal.brain-map.org/) has created opportunities for understanding how spatial variations on the molecular transcriptomic scale relate to the macroscopic neuroimaging phenotypes [64]. This will allow us to better understand the relation of imaging features to molecular substrates in brain tissue.

## What will cognition, emotion, motivation and related mental processes do for brain health in the next 20 years?

To increase the impact of molecular science for brain health, the knowledge gap between the molecular processes and cognitive and behavioral manifestations needs to be filled.

The dementia concept often used in this context is imprecise. It will be a strength to substitute with refined, objective cognitive and behavioral variables. The six cognitive domains recognized in DSM-5 are: complex attention, executive function, learning and memory, language, perceptual-motor control, and social cognition. These need to be subdivided and specified, and their associations to mood and behavior clarified, enabling studies to reveal to reveal hidden mental manifestations and associations to anatomical brain networks and biochemical processes. For instance, memory and executive components and how they are represented should be specified. Without association studies between molecular information and detailed cognitive entities, there is a risk that the success of the Brain Health project will be compromised. This is most apparent in countries where high-tech methods are not always available. Brain health is influenced by our current mental state and overall mental health status, factors that too often go unrecognized as mediators of brain health [1]. Engagement of the community at-large through public health means is needed to call attention to the importance of mental health and other less recognized factors in the maintenance of a healthy brain [65].

## The pharma industry perspective on brain health in the next 20 years

In 2006 the drug development timescale for a new drug molecule to enter the market was 10–15 years [66]. This is changing following the COVID19 epidemic, with regulatory authorities more flexible in their handling of new, innovative therapies. In the area of brain disease, in particular dementia, clinical diagnosis is still hampered by suboptimal diagnostic criteria for neurological and psychiatric disorders [67] and by lack of access to primary care and brain imaging facilities. Nevertheless, there is an aspiration that emerging therapeutic themes (neuroinflammation, neuroimmune therapies) [68] will offer significant advances in palliative and potential disease-modifying therapy in a number of disease areas, including dementia.

Over the last few decades, the pharmaceutical industry has relied heavily on academia and the biotech industry revolution to restock their pipeline for innovative drugs across a range of therapeutic areas, though investment in the CNS has been limited. Despite progress in molecular science, the factors that drive the drug industry's investment decisions have not greatly changed [67]. In the CNS area, there are several reasons why pharmaceutical companies may not be heavily invested.

1. Complexity of the condition. CNS diseases involve intricate mechanisms and pathways in the brain, making them challenging to fully understand and target effectively with drugs. Also, CNS conditions often have some disease heterogeneity. Current paucity of biomarkers is also a constraint, as is limited direct access to tissue biospecimens (compared to peripheral diseases). Regulatory frameworks forbid biopsy for most CNS disorders.
2. High research and development costs. Developing drugs for CNS disease requires substantial investment in research, clinical trials, and regulatory approval processes. The complex nature of these disorders, coupled with the need for longer-term clinical trials to detect efficacy, contribute to the high costs involved.
3. Existing treatment options. The availability of already licensed treatments may discourage companies from investing heavily in a disease area, due to existing competition and current standard of care.
4. Regulatory challenges. Regulatory requirements for drug approval in CNS disorders are especially stringent, further adding to the complexity and cost of developing new drugs. From a company fiscal viewpoint, these regulatory challenges may be barriers to investment, although new accelerated pathways exist.

In summary, investment decisions in the pharmaceutical industry are driven not only by innovative drug discoveries or advancements in scientific understanding, but also by market forces and time-dependent commercial demands. Past, present and future Nobel prizes are unlikely to unravel that true-ism.

## Final words

We have tried to foretell the future. Time will inevitably prove us wrong. There undoubtedly will be molecular breakthroughs for brain health by 2043, though they may come from fields we have mentioned only in passing, or ignored. Safe bets are likely to be found in the cellular hygiene processes (autophagy, ubiquitination), targeted manipulation of gene expression (CRISPR, DNA repair, RNAi) and novel drug discovery, all accelerated by the booster rockets of AI.

We note that many of the Nobel laureates listed in Table 3 were initially dismissed or greeted with skepticism. At least one (Shechtman, 2011) was fired because of his data. The motto "Today's weirdness is tomorrow's reason why" is pinned to many laboratory walls around the world. It is attributed to Hunter S. Thompson (1937–2005), an American journalist born in Louisville, Kentucky, best known for his book “*Fear and Loathing in Las Vegas*”. He had minimal experience of laboratory science as far as we know, or of translational medicine. But he hit on a quirky phrase that is likely to remain true, for the next 20 years and beyond.

## CRediT authorship contribution statement

**Atticus H Hainsworth:** Writing – review & editing, Writing – original draft, Visualization, Project administration, Conceptualization. **Thomas P Blackburn:** Writing – review & editing, Methodology. **Elizabeth M Bradshaw:** Writing – review & editing, Resources, Investigation. **Fanny M Elahi:** Writing – review & editing, Supervision. **Philip B Gorelick:** Writing – review & editing, Project administration, Investigation, Conceptualization. **Jeremy D Isaacs:** Writing – review & editing, Investigation, Conceptualization. **Anders Wallin:** Writing – review & editing, Project administration, Conceptualization. **Steven CR Williams:** Writing – review & editing, Validation.

## Declaration of competing interest

Hainsworth leads MRC-Dementias Platform UK Vascular Experimental Medicine group and was Chief Investigator on the PASTIS trial. He has received honoraria from Eli Lilly and NIA and serves as a scientific consultant for Aribio Inc.

Blackburn is a Non-Executive Board Member of Umecrine Cognition AB.

Bradshaw holds a role within ISTAART Immunity and Neurodegeneration PIA and is a Founder of IMAD Therapeutics.

Isaacs was Principal Investigator on the PASTIS trial. He has been a PI on clinical trials funded by Roche, Merck and Lupin Pharmaceuticals and has received funds from Nestle, Biogen and Roche.

All other authors report no conflicts of Interest related to this manuscript.

All the claims stated here come from the various authors’ personal opinions and reflections. The text in this article is in no sense official or legally binding.
